# Non-pharmacological interventions for sleep disturbance (“insomnia”) in patients with advanced cancer: a scoping review

**DOI:** 10.1007/s00520-025-09550-2

**Published:** 2025-05-21

**Authors:** Shauna Munir, Michael Connolly, Andrew Neil Davies

**Affiliations:** 1 Our Lady’s Hospice & Care Services (Community Palliative Care), Dublin, Ireland; 2 University College Dublin School of Nursing, Midwifery and Health Systems, Dublin, Ireland; 3 School of Medicine, Trinity College Dublin, Dublin, Ireland; 4 School of Medicine, University College Dublin, Dublin, Ireland

**Keywords:** Sleep disturbance, Cancer, Oncology, Palliative care, Non-pharmacological

## Abstract

**Purpose:**

Sleep disturbances are a common yet overlooked symptom in patients with advanced cancer. Pharmacological interventions have been widely used to manage sleep disturbances; however, concerns related to their adverse effects have resulted in a need for alternative interventions. The purpose of this scoping review is to appraise the published literature on the non-pharmacological management of sleep disturbances in patients with advanced cancer.

**Methods:**

This scoping review was completed using the recommended methodological framework. An extensive literature search was completed using five electronic databases (Medline, CINAHL, Embase, APA PsycINFO, CENTRAL) and the Cochrane library. There were no restrictions applied to the search in relation to the years published. Only studies published in the English language were included.

**Results:**

We identified 9 studies published between 2010 and 2023 which focused on non-pharmacological management of sleep disturbances in patients with advanced cancer. Specific interventions included cognitive behavioural therapy, mindfulness-based therapies, bright light therapy, sleep hygiene, and other non-standard interventions. Improvements in sleep disturbance were noted in all studies, but only some studies showed statistical significance.

**Conclusion:**

This scoping review identified a relatively small number of relevant studies, involving a relatively small number of participants. Moreover, the studies involved very different interventions, with very different methodologies (including type/time of assessments). Thus, it is difficult to recommend any particular non-pharmacological intervention at this time. Nevertheless, non-pharmacological interventions undoubtedly have a role to play in the management of sleep disturbance in patients with advanced cancer.

## Introduction

The International Classification of Sleep Disorders (ICSD) divides sleep disorders into six major groups: insomnia disorders, sleep-related breathing disorders, central disorders of hypersomnolence, circadian rhythm sleep–wake disorders, parasomnias, and sleep-related movement disorders [1]. Patients with advanced cancer may experience any of these disorders, although the most commonly associated disorders are insomnia (i.e., “a persistent difficulty with sleep initiation, duration, consolidation, or quality that occurs despite adequate opportunity and circumstances for sleep, and results in some form of daytime impairment” [1, 2], circadian rhythm sleep–wake disorders [3], and (to a lesser extent) sleep-related breathing disorders [4]. Importantly, many studies use the term “sleep disturbance” as a surrogate for insomnia disorders [5], and few (if any) studies use the ICSD diagnostic criteria for insomnia disorders (i.e., short-term insomnia disorder, chronic insomnia disorder).

Sleep disturbance is common in patients with advanced cancer, with a reported combined prevalence of 70.8% (95% confidence interval: 61.7–78.5) [5]. Moreover, sleep disturbance is a significant cause of morbidity in this cohort of patients [6] and is associated with a worse quality-of-life [7], as well as with a worse prognosis [8]. Importantly, sleep disturbance is also very common among the family carers of patients with advanced cancer [9]. Numerous factors have been linked to the development or persistence of sleep disturbance in cancer patients, including the underlying cancer [10], anticancer treatments [11], supportive care treatments (e.g., corticosteroids, opioids) [12], and uncontrolled symptoms (both physical and psychological) [13]. Moreover, family carers with sleep problems may be disturbing the patient (rather than vice versa).

The successful management of sleep disturbance depends on a detailed assessment (to identify precipitating/perpetuating factors for sleep disturbance), appropriate treatment, and ongoing re-assessment (to assess efficacy/tolerability of intervention). Management includes treatment of causative factors (e.g., pain, other symptoms) [14], use of non-pharmacological interventions, use of pharmacological interventions, and often a combination of these modalities [15]. A number of cancer-specific treatment guidelines have been developed [15, 16], although no analogous advanced cancer/palliative care treatment guidelines appear to have been published. Historically, pharmacological interventions have been widely used to manage sleep disturbance (i.e., benzodiazepines, “Z-drugs”). However, concerns about their usage/misuse, and especially their adverse events [17], have resulted in a greater emphasis on the use of non-pharmacological interventions [15].

Recently, the European Society for Medical Oncology (ESMO) published a Clinical Practice Guideline entitled “Insomnia in Adult Patients with Cancer” [16]. The guideline recommends the use of the following non-pharmacological interventions: (a) psychological interventions (i.e., CBT-I/cognitive behavioural therapy for insomnia, BBT-I/Brief behavioural therapy for insomnia, MBT/mindfulness-based therapy) and (b) exercise [16]. The guideline discusses bright light therapy but did not recommend this intervention (on the basis of current evidence) [16]. It should be noted that a number of other non-pharmacological interventions have been utilised for the management of sleep disturbance in patients with cancer, including other psychological techniques [18], behavioural techniques [18], exercise [19], acupuncture (and related treatments) [20], herbal medication [21], aromatherapy [22], and miscellaneous other complementary/alternative therapies.

Some of these interventions may not be suitable for patients with advanced cancer due to their impaired physical condition, cognitive problems, psychological problems, and/or their limited prognosis. Thus, the aim of this scoping review was to assess the evidence for the use of all non-pharmacological interventions in patients with advanced cancer. Importantly, the previously published systematic review of non-pharmacological interventions to manage sleep disturbance in palliative care only included studies published between 2018 and 2023, and did not include studies involving psychological interventions [23].

## Methods

The methodology utilised in this review was based on the framework developed by Arksey and O’Malley [24] but incorporated updated guidance on this framework [25]. The PRISMA Extension for Scoping Reviews (PRISMA-ScR) was used to report the outcome of this review [26]. A scoping review was undertaken due to the anticipated paucity of evidence required to undertake a systematic review and/or meta-analysis to generate “summary findings” about the effectiveness of the various non-pharmacological interventions in patients with advanced cancer [27]. Our objectives were to summarise the current evidence, identify gaps in the evidence, and highlight areas for future research.

### Search strategy

Five electronic databases (Medline, CINAHL, Embase, APA PsycINFO, CENTRAL) and the Cochrane Library were originally searched in June 2023 and re-searched in February 2025 (to check for any new references). A detailed search strategy was developed for Medline (Appendix) and adapted as needed for the other databases. There was no limit with regard to the date of publication.

### Study eligibility criteria

Inclusion criteria:

1. Studies needed to include adult patients with advanced cancer, as defined by the National Cancer Institute/NCI, USA [28]: “A term that is often used to describe cancer that is unlikely to be cured…Advanced cancer may also be used to describe cancer that has spread from where it first started to nearby tissue, lymph nodes, or other parts of the body”.
2. The focus of the studies needed to be on the management of sleep disturbance (or symptom clusters including sleep disturbance): studies exploring the generic effects of an intervention were excluded.

Exclusion criteria:

1. Studies focusing on patients with non-advanced cancer and/or receiving anticancer treatment.
2. Studies focusing on patients with non-malignant diseases.
3. Non-English studies.
4. Conference abstracts.
5. Paediatric studies.
6. Animal studies.

### Data management and synthesis

The EndNote 20™ bibliographic software (Clarivate Analytics LLP, USA) was used to store the retrieved articles, whilst the Covidence systematic review software (Veritas Health Innovation, Australia) was used to screen these retrieved articles. Two reviewers (SM, AD) independently screened the titles and abstracts for full text articles to review. A third reviewer (MC) was available to resolve potential conflicts. Two reviewers (SM, AD) independently reviewed the full text articles and extracted the relevant information using a review-specific template. A third reviewer (MC) was again available to resolve conflicts. The reference lists of all retrieved full text articles, relevant chapters in major palliative care textbooks, and relevant sections of major palliative care guidelines were hand searched for other potential studies.

Data that were extracted from the studies included;

1. Authors, year of publication, and country.
2. Population; number of participants, place of care, cancer diagnosis, age range, sex.
3. Intervention(s) used.
4. Assessments; type of study, assessments used, description of non-pharmacological intervention.
5. Results: results of the intervention, including any comments related to recruitment or withdrawal.

## Results

### Search results

**Fig. 1:**
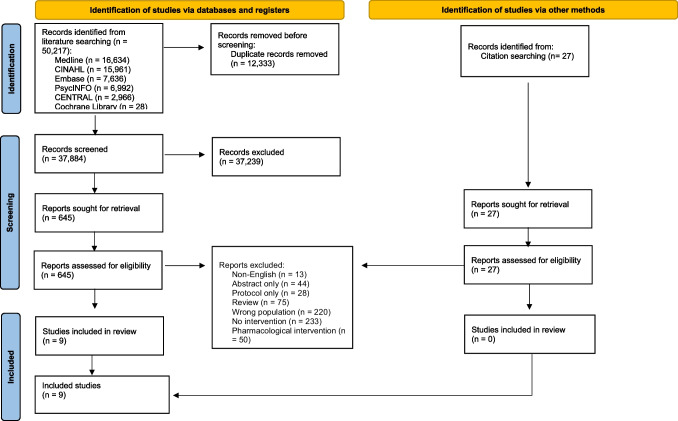
Flow chart of article inclusion

The search strategy identified 37,884 unique references, although only 672 full text articles were retrieved/reviewed (Fig. 1). Several “duplicate” records were identified among the retrieved full text articles; some were conference abstracts, some were articles reporting “early” results, and some were articles reporting different analyses/subsets of results. Nine studies evaluating various non-pharmacological interventions for sleep disturbances in patients with advanced cancer were identified from the database searches and had their data extracted [29–37]. No other studies were identified from hand searching. The studies involved a variety of non-pharmacological interventions (see Tables 1 and 2). Importantly, several studies were excluded from the scoping review because sleep was not the primary outcome of the study (and sleep disturbance was not an inclusion criterion for the study).

**Table 1:** Studies involving behavioural/psychological interventions

Reference	Population	Intervention	Assessments	Results
Bernatchez et al. (2019) [29] Canada	Outpatients with “advanced cancer” with insomnia and/or hypersomnolence *n* = 6 (insomnia, 3; insomnia and hyper-somnolence, 2; hypersomnolence, 1) Mixed cancer diagnosis Age range: 57–88 yr Female: 33% Eastern Cooperative Oncology Group score (ECOG): ECOG II, 5; ECOG III, 1	Pilot study (uncontrolled) Cognitive-behavioural and environmental intervention (CBT-E) for 3 weeks—adapted from pre-existing CBT-I intervention: combination of cognitive strategies, behavioural strategies (e.g., stimulus control), environmental strategies (e.g., noise reduction), psychoeducation, and sleep hygiene (e.g., caffeine restriction) One face-to-face session, and then telephone follow-up, with clinical psychologist: management individualised	Insomnia Severity Index (ISI) Sleep diary Actigraphy	Recruitment—“particularly difficult” Adherence—“highly variable” Withdrawals (*n* = 2)—one not included in analysis as no post intervention data Global satisfaction with CBT-E: “a lot”, 4; “moderately”, 1; no data, 1 *Results for patients with just insomnia (n* = *3):* ISI—scores improved in all patients, with 2 patients’ scores falling below the “clinically significant” threshold Sleep efficiency (sleep diary)—improved in all patients, with 2 patients moving above “poor” sleep threshold, and 1 patient remaining above this threshold (pre and post CBT-E) Sleep efficiency (actigraphy)—improved in 2 patients and deteriorated in 1 patient (but all pre and post CBT-E values were above the “poor” sleep threshold)
Wells-Di Gregorio et al. (2019) [30] USA	Outpatients with “advanced cancer” with sleep difficulties 54% patients in intervention arm had “local disease” or were in “remission” *n* = 28 (intervention arm, 17) Mixed cancer diagnosis: haematological, 26%; breast, 25%; gynaecological, 25% Age: mean 56.54 yr (SD ± 8.06 yr) Female: 82%	Pilot study/RCT (control arm was waitlist) Cognitive behavioural therapy with acceptance and commitment therapy (CBT-ACT) for 6 weeks. CBT components: relaxation (CD provided), sleep hygiene, sleep restriction; constructive worry, problem solving, goal setting, activity pacing, and behavioural activation. ACT components: perspective taking, identifying values, committed action Two face-to-face sessions, and one DVD session, then telephone follow-up, with postdoctoral fellows in psychosocial oncology	ISI—only severity items utilised Sleep Diary	Withdrawals (*n* = 5) ISI—mean scores improved in intervention arm (*p* = 0.0047) Sleep diary—mean sleep latency decreased by 14.85 min in intervention arm (*p* = 0.0062), and sleep efficiency increased by 9% in intervention arm (*p* = 0.0062)
Kwekkeboom et al. (2018) [31] USA	Outpatients with “advanced cancer” with sleep disturbance, pain, and fatigue (symptom cluster), and receiving chemotherapy *n* = 164 (intervention arm, 85) Mixed cancer diagnosis: breast, 23%; lung, 21%; gastrointestinal, 21%; gynaecological, 17% Age: mean 59.7 yr (SD ± 9.6 yr) Female: 72%	Randomised controlled trial/RCT (control arm was “cancer education”)—follow-up to pilot study [29] Cognitive behavioural strategies (CBS) for 9 weeks: education/educational booklet, together with symptom focussed imagery exercises, relaxation exercises, nature imagery exercises, nature sound recordings (via MP3 player) One face-to-face session, and then telephone follow-up, with trained therapist: management individualised/patient determined	Composite of validated tool numerical rating scales/verbal rating scales (of three symptoms)	Withdrawals (*n* = 37) Symptom cluster severity—no difference between groups at any time point (weeks 3, 6, and 9) Symptom cluster distress—less distress in intervention arm at week 6 (*p* = 0.04), but not at other time points Symptom cluster interference—no difference between groups at any time point
Kwekkeboom et al. (2012) [32] USA	Outpatients with “advanced cancer” with sleep disturbance, pain, and fatigue (symptom cluster), and receiving chemotherapy and/or radiotherapy *n* = 86 (intervention arm, 43) Mixed cancer diagnosis: gynaecological, 42%; lung, 29%; urological (prostate), 17%; gastrointestinal (colorectal), 12% Age: mean 60.29 yr (SD ± 11.09 yr) Female: 59%	Pilot study/RCT (control arm was waitlist)—follow-up to feasibility study [28] Cognitive behavioural (CB) intervention for 2 weeks: education/educational booklet, together with symptom focussed imagery exercises, relaxation exercises, nature imagery exercises, nature sound recordings (via MP3 player) One face-to-face session, and then telephone follow-up, with research nurse: management individualised/patient determined	Composite of validated tool numerical rating scales/verbal rating scales (of three symptoms)	Withdrawals (*n* = 8) Symptom cluster severity—lower in intervention group (*p* < 0.05) Sleep disturbance severity—no difference between groups Symptom cluster interference—no difference between groups
Kwekkeboom et al. (2010) [33] USA	Outpatients with “advanced” cancer with sleep disturbance, pain, and fatigue (symptom cluster), and receiving chemotherapy and/or radiotherapy *n* = 30 Mixed cancer diagnosis: gynaecological, 50%; lung, 27%; gastrointestinal (colorectal), 20%; urological (prostate), 3% Age: mean 56.27 yr (SD ± 11.23 yr) Female: 80%	Feasibility study Cognitive behavioural (CB) intervention for 2 weeks: education/educational booklet, together with symptom focussed imagery exercises, relaxation exercises, nature imagery exercises, nature sound recordings (via MP3 player) One face-to-face session, and then telephone follow-up, with research nurse: management individualised/patient determined	Composite of validated tool numerical rating scales/verbal rating scales (of three symptoms)	Withdrawals (*n* = 3) Sleep disturbance severity—no difference between groups Symptom cluster interference—no difference between groups

**Table 2:** Studies involving other interventions

Reference	Population	Intervention	Assessments	Results
Celik et al. (2023) [34] Turkey	Inpatients (hospital palliative care unit) with “cancer” with poor sleep quality (PSQI total score ≥ 5) and fatigue *n* = 52 (intervention arm, 26) Mixed cancer diagnosis: gastrointestinal, 36.54%; urological, 17.31%; head and neck, 15.38%; breast, 13.46% Age: < 57 yr, 50%; > 57 yr, 50% Female: 50%	RCT (control arm was dim red light) Bright light therapy (10,000 lx) for 30 min every morning after participant’s wake-up-time (07.00–10.00) for 2 weeks	Pittsburgh Sleep Quality Inventory/PSQI “Smart wristbands” to monitor sleep times	Statistically significant (*p* < 0.001) improvement in PSQI total score at day 14 (and day 28) in intervention arm; statistically significant difference compared to control group (*p* = 0.026) Statistically significant (*p* < 0.001) increase in total sleep time at day 14 (and day 28) in intervention arm; no statistically significant difference compared to control group (*p* = 0.259)
Hasuo et al. (2023) [35] Japan	Outpatients with “incurable cancer” with sleep disturbance *n* = 50 (intervention arm, 25) Mixed cancer diagnosis: gastrointestinal, 44%; head and neck, 14%; lung, 14% Age: mean 66.4 yr (SD ± 12.5 yr) Female: 40%	RCT (control arm was standard care) Home based heart rate variability biofeedback (HRV-BF) with resonant breathing for 5–30 min before bedtime for 5–7 days (after 5–7 days with nothing)	PSQI (Japanese version) Actigraphy	Statistically significant (*p* < 0.001) improvement in PSQI total score at day 10–14 in intervention arm; statistically significant difference compared to control group (*p* = 0.017) Statistically significant improvement in most actigraphy parameters (total sleep time, sleep efficiency, wake time after sleep onset, and number of awakenings) at day 10–14 in intervention arm; statistically significant difference compared to control group for those actigraphy parameters
Yennurajalingam et al. (2021) [36] USA	Outpatients with “advanced cancer” with poor sleep quality (PSQI total score ≥ 5), and receiving cancer therapy *n* = 67 (intervention arm, 17) Mixed cancer diagnosis: breast, 29.7%; gastrointestinal, 25.0%; gynaecological, 12.5% Age: not stated Female: 66%	Feasibility study/RCT—eight arms Eight arms involved combinations of bright light therapy (or dim red light ≈ “placebo”), and melatonin (or placebo tablet), and methylphenidate (or placebo tablet) for 2 weeks. Participants also received three psycho-educational sessions on sleep education, cognitive strategies (targeting negative thoughts), and relaxation training (CD provided)	PSQI Insomnia severity index (ISI) Actigraphy	Withdrawals (*n* = 13) Bright light therapy alone: statistically significant (*p* < 0.05) improvement in PSQI total score at day 15 (but not day 29) Bright light therapy and melatonin: statistically significant (*p* < 0.05) improvement in PSQI total score at day 15 (and day 29). Improvement is greater than bright-light therapy alone ISI—no difference between groups at any time point Actigraphy—no difference between groups at any time point
Ducloux et al. (2012) [37] Switzerland	Inpatients (Division of Palliative Medicine) with “advanced cancer” with sleep disorder (defined according to International Classification of Sleep Disorders) *n* = 18 (intervention arm, 9) Mixed cancer diagnosis: breast, 27.8%; lung, 22.2%; gastrointestinal, 11.1%; urological, 11% Age: mean 63.5 yr Female: 67%	Pilot study/RCT (control arm was waitlist) Study lasted 9 days. “Immediate intervention group” received intervention from day 3–6, whilst “delayed intervention group” received intervention from day 6–9. Intervention involved one to one relaxation session (deep breathing exercises, somatic tension release), and audio recording of training programme to listen to at night-time	Numerical rating scale (“satisfaction” with sleep) Sleep diary	Withdrawals (*n* = 7) Satisfaction with sleep improved in a similar manner in both groups at all time points (day 2, day 5, and day 9)

### Summary of included studies

The studies were mostly small in size (median, 50 participants; range, 6–164 participants; pooled number, 501 participants) and involved a diverse variety of interventions (see below). Seven were randomised controlled trials (RCTs) [30–32, 34–37], and six were labelled as feasibility, pilot, or “preliminary” studies (including four RCTs) [29, 30, 32, 33, 36, 37]. All studies involved subjective assessment (i.e., patient reported outcome measures), with five studies using validated sleep assessment tools: three studies used the Pittsburgh Sleep Quality Inventory/PSQI [34–36], three studies used the Insomnia Severity Index/ISI [29, 30, 36], with one study using both PSQI and ISI [36]. However, only four studies involved objective assessment of sleep parameters (i.e., actigraphy, “smart wristbands”) [29, 34–36]. Five studies were undertaken in the United States [30–33, 36], and one in Canada [29], Turkey [34], Japan [35], and Switzerland [37].

### Interventions

#### CBT-I/cognitive behavioural therapy for insomnia and related interventions

CBT-I is a well-established intervention for managing chronic insomnia in the general population (“moderate” quality of evidence) [18], and appears to be effective in different cohorts of cancer patients [38]. CBT-I is a multimodal intervention, which includes sleep restriction, stimulus control, sleep hygiene, relaxation techniques, and cognitive therapy (aka. cognitive restructuring): it can be administered face to face (individually, group therapy) or remotely (video, on-line). The search of the literature identified a single study of a CBT-I-related intervention in patients with advanced cancer [29], and four studies of other CBT-related interventions in this cohort of patients (see Table 1) [30–33]. Wells-DiGregorio et al. (2019) [30] reported a statistically significant improvement in ISI scores and sleep diary parameters (sleep latency, sleep efficiency) with their CBT-based intervention. Bernatchez et al. [29] reported similar findings in their study, but also reported difficulty in recruitment and high variability in adherence to the intervention. The other (related) studies focused on the symptom cluster of sleep disturbance, pain, and fatigue [31–33], and the definitive RCT reported less distress following the intervention (but not less severity or interference) [31]. CBT-I is not regularly utilised within palliative care (or indeed known about by palliative care professionals) [39].

#### Sleep hygiene

Sleep hygiene consists of “a set of general recommendations about lifestyle (e.g., diet, exercise, substance use) and environmental factors (e.g., light, noise, temperature) that may promote or interfere with sleep” [18]. Sleep hygiene may include some education about what constitutes “normal” sleep. Sleep hygiene is an integral aspect of CBT-I and, otherwise, is often used in combination with sleep restriction therapy and stimulus control. The quality of evidence for the use of sleep hygiene in generic patients with chronic insomnia is “low” [18], and evidence for its use in patients with advanced cancer is limited to a single abstract [40].

#### Bright light therapy

Currently, bright light therapy is not recommended in patients with cancer due to the paucity of evidence [16]. Nevertheless, there is some positive evidence involving patients with advanced cancer (see Table 2) [34, 36]. Celik et al. [34] (2023) reported a statistically significant improvement in PSQI scores at day 14 (end of treatment) and also at day 28, as well as a statistically significant increase in total sleep time. Similarly, Yennurajalingam et al. [36] reported a statistically significant improvement in PSQI scores at day 15 (end of treatment) in patients treated with bright light therapy alone (and bright light therapy with melatonin). However, there was no difference in ISI scores or actigraphy parameters, and the improvement in PSQI scores was not maintained (at day 29).

#### Exercise therapy

A recent systematic review confirms that aerobic exercise (e.g., walking, cycling) and mind–body exercise (e.g., qigong, tai chi, yoga) improve sleep disturbance in symptomatic patients with cancer [19]. However, it appears that there are no analogous studies involving symptomatic patients with advanced cancer. Nevertheless, non-sleep-focused studies suggest that aerobic exercise and mind–body exercise can improve sleep in this cohort of patients (patients with advanced cancer) [19, 41–45].

#### Other behavioural/psychological

A number of other behavioural/psychological interventions have been used to manage sleep disturbance in the general population (e.g., relaxation, biofeedback) [18]. Relaxation therapies include strategies to reduce somatic tension and/or cognitive arousal. Evidence for its use in generic patients with chronic insomnia is rated as “very low” [18], and we identified a single (negative) study involving patients with advanced cancer (see Table 2) [37]. Biofeedback is a variant of relaxation therapy that employs a device that monitors/informs about an aspect of the person’s physiology (e.g., heart rate, respiratory rate). Evidence for its use in generic patients with chronic insomnia is equally rated as “very low” [18], and we identified a single (positive) study involving patients with advanced cancer (see Table 2) [35]. Hasuo et al. (2023) [35] reported that heart rate variability biofeedback was associated with a statistically significant improvement in PSQI total score, as well as statistically significant improvement in certain actigraphy parameters (i.e., total sleep time, sleep efficiency, wake time after sleep onset, number of awakenings) at the end of the study.

#### Miscellaneous (non-standard) interventions

Various other interventions have been reported to be effective in the management of sleep disturbance, with the following ones reported in patients with advanced cancer (or cohorts including patients with advanced cancer): (a) eye movement/oculomotor training [46], (b) music therapy [47], (c) (Chinese) five-element music therapy [48], (d) massage (for symptom cluster of pain, fatigue, and sleep disturbance) [49], and (e) cranial electrotherapy stimulation (for different symptoms including sleep disturbance) [50]. Unsurprisingly, the evidence to support these interventions is somewhat limited (and generally comprises a single report).

## Discussion

This unique scoping review highlights the paucity of evidence for non-pharmacological interventions for the management of sleep disturbance in patients with advanced cancer. Moreover, the quality of the identified evidence is somewhat limited. Indeed, currently, it would be difficult to justify recommending any non-pharmacological interventions for the management of sleep disturbance in patients with advanced cancer. Non-pharmacological interventions are well established in other areas of clinical practice [18], and there is good evidence to support many of them in patients receiving anticancer treatment, and those that have completed their anticancer treatment (and are “living beyond cancer”) [16]. However, these cohorts of patients are very different from patients with advanced cancer, and so it is inappropriate to extrapolate the results of studies done in these cohorts of patients (to patients with advanced cancer). Nevertheless, non-pharmacological interventions undoubtedly have a role to play in the management of sleep disturbance in patients with advanced cancer.

The scoping review identified a number of studies involving CBT-related interventions. However, the treatment regimens were very different, and there is a need for standardisation in future studies (with the focus on the “gold standard” of CBT-I). Indeed, standardisation of methodology is essential for all future studies, and this includes the assessment of the sleep disturbance (subjective, objective). Moreover, studies need to employ validated “cut-offs” for determining “clinically meaningful differences”—many of the identified studies reported statistically significant improvements, but it was unclear whether these were clinically meaningful improvements. Importantly, the scoping review did not identify any studies involving certain recommended non-pharmacological interventions, i.e., BBT-I, MBT, and exercise [16]. Studies of these modalities would appear to be appropriate to undertake in this cohort of patients. Importantly, many of the included studies were small, and future studies need to be larger (and so adequately powered). Finally, there needs to be standardisation of the reporting of studies, and particularly of participant characteristics (to determine relevance).

In conclusion, non-pharmacological interventions are becoming the standard for the management of sleep disturbance in the general population. However, there is a paucity of evidence for their acceptability, efficacy, and tolerability in patients with advanced cancer. Further research needs to be undertaken to address these questions and to support the adoption in routine clinical practice.

## Data Availability

No datasets were generated or analysed during the current study.

## Appendix

### Medline search strategy

1. Sleep – MeSH

***or 2-23***

2. Sleep deprivation – MeSH
3. Sleep disorders, circadian rhythm – MeSH
4. Sleep duration – MeSH
5. Sleep initiation and maintenance disorders – MeSH
6. Sleep latency – MeSH
7. Sleep quality – MeSH
8. Sleep wake disorders – MeSH
9. Sleep arousal disorders – MeSH
10. Sleep disorders, intrinsic – MeSH
11. Insomnia – Key word
12. Sleep disturbance – Key word
13. Sleep problem – Key word
14. Sleep hygiene – MeSH
15. Sleep aids, pharmaceutical – MeSH
16. Hypnotics and sedatives – MeSH
17. Melatonin – MeSH
18. Phototherapy – MeSH
19. Light therapy – Key word
20. Cognitive behavioural therapy – MeSH
21. CBT-I – Key word
22. Complementary therapies – MeSH
23. Alternative therapies – Key word

***and***

24. Neoplasms – MeSH

***Or***

25. CANCER – Key word
